# Christian Orthodox Fasting as a Traditional Diet with Low Content of Refined Carbohydrates That Promotes Human Health: A Review of the Current Clinical Evidence

**DOI:** 10.3390/nu15051225

**Published:** 2023-02-28

**Authors:** Constantinos Giaginis, Maria Mantzorou, Sousana K. Papadopoulou, Maria Gialeli, Andreas Y. Troumbis, Georgios K. Vasios

**Affiliations:** 1 Department of Food Science and Nutrition, School of Environment, University of the Aegean, 81400 Myrina, Greece; 2 Department of Nutritional Sciences and Dietetics, School of Health Sciences, International Hellenic University, 57001 Thessaloniki, Greece; 3 Department of Environment, School of Environment, University of the Aegean, 81100 Mytilene, Greece

**Keywords:** Christian Orthodox fasting, low-carbohydrate diet, cardiometabolic health, mental health, refined carbohydrates, public health, weight control

## Abstract

Introduction: Christian Orthodox fasting is a pattern high in complex carbohydrates and low in refined carbohydrates. It has been explored in association with its potential health benefits. The present review aims to comprehensively explore the existing available clinical data concerning the potential favorable impact of the dietary pattern of Christian Orthodox fasting on human health. Methods: PubMed database, Web of Science and Google Scholar were extensively searched in order to identify the more appropriate clinical studies that explore the effect of Christian Orthodox fasting on health-related outcomes in humans utilizing relative keywords. We initially retrieved 121 records through database searching. After applying several exclusion criteria, 17 clinical studies were finally included in this review study. Discussion: Christian Orthodox fasting showed beneficial effects concerning glucose and lipid control, whereas the data for blood pressure remain inconclusive. Concerning weight control, fasters were characterized by lower body mass and lower caloric intake in the course of the fasting periods. During fasting, this pattern is higher in fruits and vegetables, showing the absence of dietary deficiencies for iron and folate. Nevertheless, dietary deficiencies were recorded for calcium and vitamin B2, and also hypovitaminosis D has been noticed in monks. Interestingly, the vast majority of monks do present with both good quality of life and mental health. Conclusions: Overall, Christian Orthodox fasting is a dietary pattern low in refined carbohydrates and high in complex carbohydrates and fiber that may be beneficial for human health promotion and chronic disease prevention. However, further studies are strongly recommended on the impact of long-term religious fasting on HDL cholesterol levels and blood pressure.

## 1. Introduction

Orthodox fasting lasts for a total of 180–200 days each year, as imposed by the Christian Orthodox religion. There are three main fasting periods, with different foods being prohibited during each fasting period. During the 40-day Nativity fast, fasters avoid dairy, eggs, fish and meat. In addition, fasters abstain from olive oil on Wednesdays and Fridays. During Lent, which lasts for 48 days, fasters abstain from dairy products, eggs, and meat. Additionally, fasters also abstain from olive oil on weekdays and from fish every day, except for 25 March and Palm Sunday [1,2]. During the 15-day Assumption fasting, fasters abstain from dairy products, eggs, and meat. Also, fasters abstain from olive oil on weekdays and from fish every day except for 6 August. Furthermore, every Wednesday and Friday, fasters abstain from dairy, eggs, fish, meat, and olive oil. During the week immediately following Christmas, Easter, and the Pentecost, fasting prohibitions do not take place [1,2]. As a result, the fasters are led to a dietary pattern that includes fruits, vegetables, legumes, seafood (apart from fish on most fasting days), whole grains and nuts, which are rich in complex carbohydrates and fiber and low in refined carbohydrates [1,2,3,4,5]. Regarding macronutrient intakes during fasting, fasters report lower energy, fat and saturated fat intakes and increased complex carbohydrate and fiber intakes, while protein intake varies [6]. Overall, during the fasting period, fasters abstain from delicacies that are usually high in refined carbohydrates due to religious fasting’s dietary prohibitions and the aim to abstain from highly palatable foods [1,2,3,4]. The exact ratios of macronutrients during fasting do vary between people, according to their personal preferences, while monks do follow more strict dietary rules throughout their life and during fasting [1,2,3,4].

However, Orthodox Christians that do fast are likely to follow various different religious fasting practices and may fast for different time periods, as well [3]. Different styles of fasting have been investigated regarding their impact on human health. Caloric restriction, alternate-day fasting, and dietary restriction, as is the Christian Orthodox fast, are the most common ones [3,4,5]. Positive health effects on chronic disease in humans and animal models have been described when fasting [1,2]. Christian Orthodox fasting has been compared to the Mediterranean Diet and is deemed to be an integral part of this dietary pattern [4] as well as a form of periodic vegetarianism [5]. The Mediterranean Diet is the best-studied dietary pattern in relation to cardiometabolic health, a pattern high in complex carbohydrates and low in added sugars, with health benefits regarding both prevention and management of chronic non-communicable diseases [6]. In fact, Christian Orthodox fasting practices also adhere to the World Cancer Research Fund’s Cancer Prevention Recommendations due to the high intakes of fiber-rich foods, low intakes of refined carbohydrates and low consumption of meat products [6]. WHO advises a low intake of free sugars for both adults and children and an intake of refined sugars limited to less than 10% of total energy intake, with further benefits when reduced to less than 5% of the daily energy intake [7]. Notably, high intakes of refined sugars, especially as part of a “Western diet” pattern, is associated with a greater risk of chronic diseases, such as cardiovascular disease [8], dyslipidemia [9,10,11,12], metabolic syndrome [12], and ectopic fat accumulation [13].

Regarding carbohydrates, the dietary pattern of Greek Orthodox fasting is characterized by higher consumption of pulses, grains, vegetables, and fruits in combination with a limitation of foods of animal origin [14]. During fasting, the consumption of non-refined carbohydrates and fiber seems to increase [6]. In Athonian monks, non-refined carbohydrate intake appears to be decreased. However, this might be due to the stricter restriction that has happened in monasteries concerning both variety and amount of food in studies on monks [15]. However, dietary fiber intake increases due to the intake of foods that are allowed during the fasting periods [16,17,18,19].

In view of the above considerations, the present review aims to critically collect and in-depth summarize the current clinical data concerning the potential beneficial effects of Christian Orthodox fasting on human health promotion. For this purpose, the PubMed database, Web of Science and Google Scholar were comprehensively searched by the use of relative keywords in order to identify the most appropriate clinical human studies.

## 2. Methods

**Figure 1:**
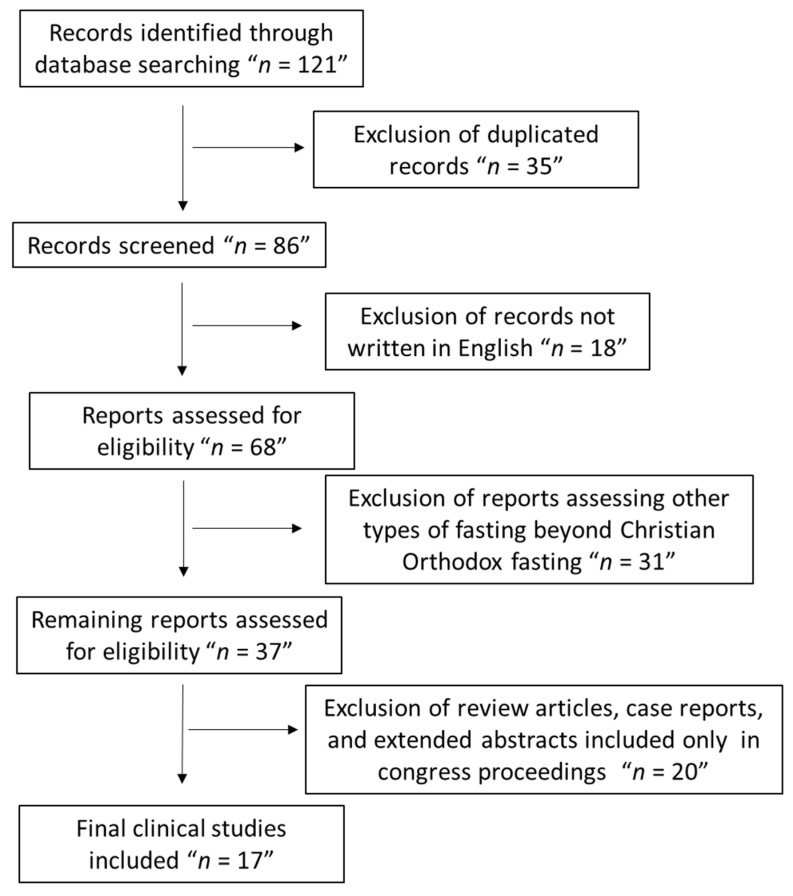
Flow chart of the study population.

This is a comprehensive review of the currently existing clinical human studies concerning the effect of Christian Orthodox fasting on health-related outcomes in humans, such as cardiovascular diseases, metabolic disorders, cognitive impairment, and health-related quality of life. In fact, the most accurate scientific databases, PubMed, Scopus, Web of Science and Google Scholar, were thoroughly searched utilizing related keywords, such as “Christian”, “Orthodox”, “fasting”, “low carbohydrates diets”, “refined carbohydrates:”, “cardiometabolic health status”, “weight control”, “nutrient intakes”, “sufficiency status”, “nutrients’ deficiencies”, “hypertension”, “obesity”, “triglycerides”, “HDL”, ” LDL”, “mental health”, “quality of life”, and “headache” to find the already existing clinical human studies, from the last 32 years (1990–2022). The results were filtered according to relevance, and the most appropriate ones were selected and analyzed below. We included only human clinical studies written in the English language that were found on PubMed, Scopus, Web of Science, and Google Scholar. We excluded studies that were not included in PubMed, Scopus, Web of Science, and Google Scholar. We also excluded studies that examined other types of fasting beyond Christian Orthodox fasting, as well as review articles, case reports, and extended abstracts included only in congress proceedings. By using the aforementioned criteria, 17 clinical studies fulfilled all insertion criteria and were chosen for this evaluation. Figure 1 depicts the research screening process in the form of a flowchart.

## 3. Results

### 3.1. Cardiometabolic Health Status

**Figure 2:**
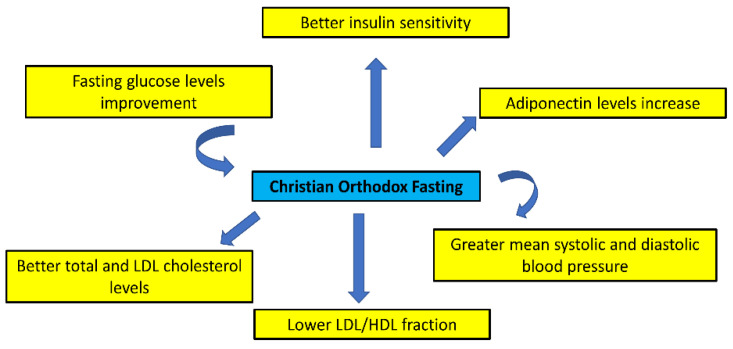
Potential benefit effects of Christian Orthodox fasting on cardiometabolic factors.

Several studies currently investigate the impact of Christian Orthodox fasting on glycemia (Table 1, Figure 2). A recent study showed the benefit of religious fasting on fasting glucose levels [20]. A previous cross-sectional study examined the effects of Christian Orthodox fasting on cardiometabolic biomarkers in 50 Athonian monks (38.7 ± 10.6 years old). Both lipid and glucose indices, as well as the homeostasis model assessment of insulin resistance (HOMA-IR), were found to be within the normal range [15]. A following study also compared the diet of 43 males from the general population that regularly fast (20–45 years) and 57 age-matched Athonian monks [16]. Monks had better insulin sensitivity, as assessed by HOMA-IR, compared to the general public [16]. Accordingly, during Christmas fasting, a significant decrease in glucose levels was reported after the fasting period [5]. However, it should be noted that no changes in fasting glucose were noted during a one-year period or during a 40-day fasting period [17,18]. Another study on the effect of Orthodox fasting on adiponectin levels showed that those who fasted increased their adiponectin levels during the fasting period, an effect that has metabolic advantages for human health [21]. Fasting might ameliorate glucose control, yet further studies are needed to draw firm conclusions. Moreover, the implementation of Christian Orthodox fasting improved plasma adiponectin concentrations compared with time-restricted eating in overweight premenopausal women. However, this positive effect cannot be generalized to other population groups [21].

The role of religious fasting on lipid control in people with and without dyslipidemia has recently been investigated. A recent study showed that Christian Orthodox fasting might result in reductions in HDL, LDL, and triglyceride levels, especially in people with dyslipidemia [20]. In this respect, a previous pilot study was performed on 10 Greek Christian Orthodox monks aged 25–65 years, with BMI > 30 kg/m^2^, that lived in two monasteries in Crete during a fasting week and compared them to their normal diet. Measurements were taken during Palm Sunday week (fasting) and the week after Pentecost Sunday (non-fasting) [19]. Interestingly, the blood lipid profile was improved during the fasting week. Specifically, total and LDL cholesterol levels were considerably better at the end of the non-fasting week, and a non-significant increase in HDL cholesterol was noted [19]. During the fasting week, the fraction of total to HDL cholesterol was decreased, but serum triglycerides were increased [19].

Fasting may considerably contribute to the observed favorable biomarker profiles in a population group that fast for 24.4 (±10.4) years [19]. Considering the impact of Christian Orthodox fasting on serum lipoproteins, it has been reported that at the end of the fasting period, fasters exhibited 12.5% lower total cholesterol and 15.9% lower LDL cholesterol compared to non-fasters. The LDL/HDL fraction was lower for fasters, but the alteration in HDL cholesterol was not statistically significant [17]. Similar findings were observed when the pre-fasting and post-fasting values of fasters were compared. Nevertheless, no differences were found in non-fasters [15]. Additionally, in a 40-day (fasting and non-fasting days) survey on 36 nuns and monks, a reduction in nutritional and plasma cholesterol, a rise in triglycerides and a moderate drop in LDL/HDL during fasting were noted [18].

A recent study of 60 healthy overweight Greek adults was conducted to compare the effects of Christian Orthodox fasting and time-restricted fasting on blood lipids over a period of seven weeks, indicating considerable decreases in overall HDL and LDL cholesterol [22]. Overall cholesterol and HDL cholesterol decreases were more pronounced during Christian Orthodox fasting compared to time-restricted fasting, in spite of similar anthropometric indices [22]. In another study, positive impacts on overall and LDL cholesterol have been found, yet the effect on HDL cholesterol remains unclear [23]. Subjects were not on lipid control medicines. Hence, studies on fasters under lipid-lowering drugs may have dissimilar findings, while the impact of fasting on blood lipids may not be evident six weeks after the end of the seven-week fasting period.

Certain studies have explored whether Christian Orthodox fasting may affect blood pressure values in those who fast. In this respect, blood pressure changes of 38 devout Christian Orthodox fasters and 29 matched controls living in Crete were monitored for one year. Data were gathered before and at the end of the three major fasting periods of the Christian Orthodox calendar (Christmas, Easter, and Assumption) [24]. During the course of this survey, fasters exhibited greater mean systolic and diastolic blood pressure (SBP and DBP, respectively) compared to the control group, while the non-fasting period had a substantial effect in decreasing the blood pressure levels of fasters [24]. Towards the end of fasting periods, fasters’ prevalence of Christmas and Lent “high-normal” blood pressure was greater compared to that of the controls, while it was lowered during the Assumption and reached the very low levels of controls. Blood lipids were considerably related to SBP/DBP at most measurements [24]. However, in another study, systolic, but not diastolic, blood pressure was considerably greater, while the Christian Orthodox religious fasting diet did not appear to lead to an observable impact on blood pressure [19].

In addition, it should be noted that water-only fasting may reduce body weight, blood pressure, and lipolytic activity of fasting hypertensive patients without affecting the average heart rate. Interestingly, Ramadan fasting enhanced lipid profile, although it showed conflicting results for body weight, blood pressure, and heart rate variability [25]. Considering the limited studies in this field, further research should be conducted to support the clinical impact of fasting on the cardiovascular health of patients with hypertension.

**Table 1:** Studies concerning the impact of Christian Orthodox fasting on cardiometabolic factors.

Cardiometabolic Factor	Study Population	Study Period	Main Results	References
Glucose Control	Cross-sectional study in 50 Athonian monks (mean age: 38.7 ± 10.6 years)	Restrictive and non-restrictive fasting days	Glucose indices and HOMA-IR in normal levels	[15]
	43 males from the general population that regularly fast (20–45 y of age) and 57 age-matched Athonian monks	Restrictive and non-restrictive days	Monks had better HOMA-IR than the general population	[16]
	37 strict fasters (18 males, 19 females, mean age 43.0 ± 13.1 years), vs. 48 age-and sex-matched controls (21 males, 27 females; mean age 38.6 ± 9.6 years)	40 days	Significant decrease in glucose levels after the fasting period	[5]
	120 Greek adults were followed longitudinally (60 fasters, 60 non-fasters)	1 year	No changes in fasting glucose of fasters	[17]
	36 (25 women & 11 men) monks from 5 Greek monasteries	40 days	No changes in fasting glucose	[18]
Blood Lipid Control	10 Greek Christian Orthodox monks aged 25–65 years, with BMI >30 kg/m^2^	1 fasting week vs. non-fasting week (Measurements on Palm Sunday week (fasting) and the week following Pentecost Sunday (non-fasting)	End of the non-fasting week: ↑* total and LDL cholesterol NS ↑ HDL cholesterol During the fasting week: ↓ total: HDL cholesterol ↑ serum triglycerides	[19]
	120 Greek adults were followed longitudinally (60 fasters, 60 non-fasters)	1 year	↓** 12.5% total cholesterol & ↓ 15.9% LDL cholesterol in faster than non-fasters. ↓ LDL/HDL in fasters, NS change on HDL cholesterol in fasters Similar results were found when the pre- and after-fasting values of fasters were compared	[17]
	36 (25 women & 11 men) monks from 5 Greek monasteries	40 days	↓ dietary and plasma cholesterol ↑ triglycerides ↓ LDL/HDL during fasting	[18]
	37 overweight but healthy adults followed a hypocaloric diet based on Orthodox fasting 23 BMI-matched healthy adults followed a hypocaloric, time-restricted eating plan	7 weeks	Lower total cholesterol after Orthodox fasting than time-restricted fasting (178.40 ± 34.14 vs. 197.09 ± 29.61 mg/dL, *p* = 0.028) Lower HDL cholesterol after Orthodox fasting than time-restricted fasting (51.01 ± 11.66 vs. 60.13 ± 15.93 mg/dL, *p* = 0.013)	[21]
	29 overweight but healthy adults followed a hypocaloric diet based on Orthodox fasting 16 age- and weight-matched healthy adults followed a hypocaloric, time-restricted eating plan	7 weeks and 6 weeks follow up after the intervention	Total cholesterol, HDL and LDL cholesterol were reduced at 7 weeks and increased at 6 weeks after Orthodox fasting cessation	[22]
	60 Greek Orthodox participants, 30 with dyslipidemia and 30 without dyslipidemia who followed the Greek Orthodox fasting	7 weeks	In both groups: ↓ fasting glucose, ↓ HDL, ↓ LDL and ↓ triglyceride levels ↑ Hemoglobin, ↑ hematocrit, ↑ iron and ↑ ferritin levels ↓ vitamin B12 and calcium levels Better cholesterol levels improvements on people with dyslipidemia	[20]
Blood Pressure Control	38 devout Christian Orthodox fasters and 29 matched controls living in Crete	1 year (measurements before and at the end of the three major fasting periods)	Fasters had ↑ mean SBP and SBP than controls. ↓ BP during the non-fasting period Blood lipids were significantly associated with SBP/DBP at most measurements	[24]
	10 Greek Christian Orthodox monks aged 25–65 years, with BMI >30 kg/m^2^	1 fasting week vs. non-fasting week (Measurements on Palm Sunday week (fasting) and the week following Pentecost Sunday (non-fasting)	↑ SBP while fasting	[10]

↑*: Increase; ↓**: Decrease.

### 3.2. Weight Control 

**Figure 3:**
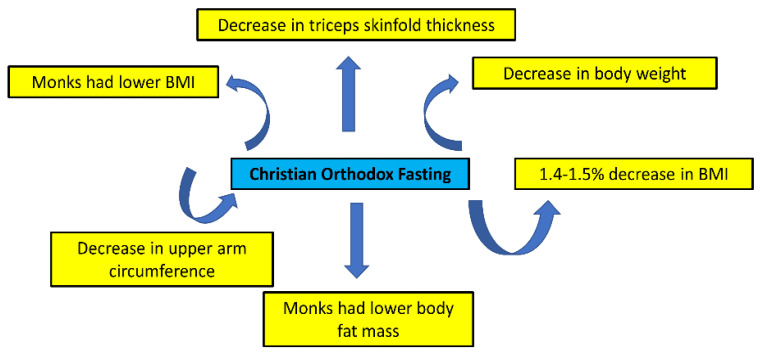
Potential benefit effects of Christian Orthodox fasting on weight control.

The impact of fasting on body mass and weight control has been investigated in four studies (Table 2, Figure 3). A recent study conducted on 43 males (20–45 years old) from the general population that regularly fasted and 57 age-matched Athonian monks found that monks had lower Body Mass Index (BMI) and lower body fat mass [16]. Moreover, a 40-day (fasting and non-fasting days) prospective study on 36 nuns and monks found that fasting led to a decrease in weight, upper arm circumference, and triceps skinfold thickness [18]. Moreover, a decrease in monks’ body mass was observed during one fasting week, although not at a significant level. Interestingly, a one-year longitudinal study found that fasters had 1.5% lower BMI than non-fasters at the end of the fasting period, while a 1.4% decline in BMI was also observed in fasters after the fasting period [17]. Hence, Christian Orthodox fasting may benefit weight control. In this respect, it should also be noted that the decrease in BMI due to fasting is very low, and thus it cannot lead to unhealthy effects in people with normal BMI.

### 3.3. Nutrient Intakes and Sufficiency Status

**Figure 4:**
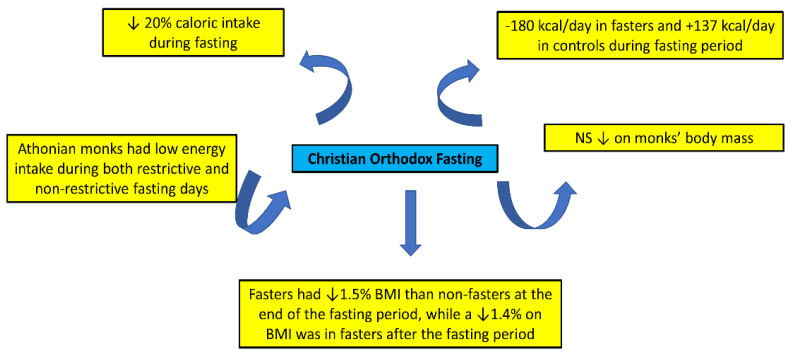
Potential effects of fasting concerning nutrient intakes and nutritional status (↓: Decrease).

Diet quality, as well as macronutrient and micronutrient intakes during Orthodox fasting, is an important issue to consider, especially when taking into account the fact that fasting takes place for 180–200 days per year. Several studies have investigated the intake and nutrient status of fasters (Table 3, Figure 4).

During fasting, caloric intake decreases. In this respect, a decrease in caloric intake during fasting by 20% has been reported [18], and fasters presented a decrease of 180 kcal energy intake, while there was an increase of 137 kcal in the controls during the fasting period [4,18]. Moreover, Athonian monks appeared to have low energy intake during both restrictive and non-restrictive fasting days, while carbohydrate and saturated fat intakes were lower, and protein was higher during the “restrictive days” [15]. In another study, 43 males from the general population that regularly fast (20–45 years old) and 57 age-matched Athonian monks were enrolled. Monks had lower daily total caloric intake for both “restrictive” and “non-restrictive days” than the general public [16,17].

Considering other macronutrient intakes during fasting, monks and fasters had lower intakes of total and saturated and trans fats [4,18], higher intakes of dietary fiber [4,19,20,21,22,23], and lower protein intake [4].

Consumption of legumes and fish/seafood is increased during fasting, and consumption of dairy products, meat and eggs increased significantly after the fasting week. Considering dietary components, fasters seem to increase fruit and vegetable consumption during the fasting periods and decrease their sodium intake compared to no fasters.

Considering micronutrient intakes, an increase in magnesium intake during fasting has been reported [24]. Certain studies have also observed higher folate intakes during fasting [4,19]. As far as iron status is concerned, a study was performed on 35 Greek Christian Orthodox strict fasters (17 male and 18 female, with a mean age of 43.6 ± 13.2 years) and 24 controls (11 male and 13 female, with a mean age of 39.8 ± 7.6 years) whose iron status was assessed before and near the end of the Christmas fasting period (meat and dairy were prohibited) [26]. Fasters had marginally worse pre-fasting hematological indicators, yet values were well above the cut-off levels, suggesting that long-term religious fasting did not negatively affect iron status [26]. Notably, during the fasting period, the changes in iron status measurements were more beneficial for fasters than for non-fasters as fasters increased their ferritin levels and decreased their total iron-binding capacity, especially those fasters who were female. No one presented with iron deficiency at the end of the fasting period [26]. At the end of the fasting, dietary iron intake was significantly higher in fasters compared to non-fasters [25]. Hence, fasting may not impact iron status and may not be associated with a significantly greater degree of iron deficiency in fasters with normal iron status. This finding was in accordance with the observed increase in iron intake by another study, which also found higher levels of iron, hemoglobin, hematocrit and ferritin levels, but low levels of vitamin B12 in fasters [19,20]. In this respect, it should be noted that the decrease in vitamin B12 levels was low and did not affect iron, hemoglobin, hematocrit, and ferritin levels, which were increased. However, fasters with low vitamin B12 levels before the fasting period may develop anemia. In this case, supplement consumption should be considered in order to avoid vitamin B12 deficiency during fasting.

Another study followed Greek Orthodox Christians from Crete for one year. Half of the subjects fasted regularly, and half did not (non-fasters) [4]. Before and near the end of fasting-period days, measurements were performed, and there were no differences for other vitamins or minerals, before and after fasting, except for vitamin B2 [4].

Karras et al. found low vitamin D levels and high parathyroid hormone (PTH) with normal serum calcium levels in 50 Athonian monks in two of their published studies [15,16]. Another study also found that calcium intake decreased during fasting, as dairy is prohibited [4,19,20,24]. However, musculoskeletal metabolism and bone density are not negatively affected by Christian Orthodox fasting [26].

**Table 3:** Studies concerning the impact of Christian Orthodox fasting on nutrient intakes and sufficiency.

Nutrients	Study Population	Study Period	Main Results	References
Energy	36 (25 women and 11 men) monks from 5 Greek monasteries	40 days	↓* 20% caloric intake during fasting	[18]
	120 Greek adults were followed longitudinally (60 fasters, 60 non-fasters)	1 year	−180 kcal/day in fasters and +137 kcal/day in controls during fasting period	[4]
	50 Athonian monks (mean age: 38.7 ± 10.6 years) and 43 males from the general population that regularly fast (20–45 y of age) and 57 age-matched Athonian monks	Restrictive and non-restrictive fasting days	Athonian monks had low energy intake during both restrictive and non-restrictive fasting days	[15,16]
Macronutrients and foods	50 Athonian monks (mean age = 38.7 ± 10.6 years)	Restrictive and non-restrictive fasting days	↓* carbohydrate and saturated fat intakes ↑** protein during the “restrictive days”	[15]
	10 Greek Christian Orthodox monks aged 25–65 years, with BMI >30 kg/m^2^	1 fasting week vs. non-fasting week	↓ intakes of total and saturated and trans fats ↑ fiber during fasting ↑ legumes and fish/seafood during fasting, ↑ dairy products, meat and eggs after the fasting week	[19]
	120 Greek adults were followed longitudinally (60 fasters, 60 non-fasters)	1 year	Fasters (vs. controls) ↓ dietary cholesterol, total fat, saturated fatty acids, trans-fatty acids and protein, and ↑ fiber at the end of the fast	[4]
	35 Greek Christian Orthodox strict fasters (*n* = 17 male, *n* = 18 female; mean age: 43.6 ± 13.2 years) and 24 controls (*n* = 11 male, *n* = 13 female; mean age 39.8 ± 7.6 years)	40 days Measurements before and near completion of the Christmas fasting	↑ fiber intake	[26]
	38 devout Christian Orthodox fasters and 29 matched controls living in Crete	1 year (measurements before and at the end of the three major fasting periods)	↑ fruit and vegetable consumption during the fasting periods	[24]
Micronutrients	38 devout Christian Orthodox fasters and 29 matched controls living in Crete	1 year (measurements before and at the end of the three major fasting periods)	↓ sodium intake ↑ magnesium intake ↑ folate intake ↓ calcium intake during fasting	[24]
	10 Greek Christian Orthodox monks aged 25–65 years, with BMI >30 kg/m^2^	1 fasting week vs. non-fasting week	↑ folate intake ↑ iron intake	[19]
	35 Greek Christian Orthodox strict fasters (*n* = 17 male, *n* = 18 female; mean age: 43.6 ± 13.2 years) and 24 controls (*n* = 11 male, *n* = 13 female; mean age: 39.8 ± 7.6 years)	40 days Measurements before and near completion of the Christmas fasting	longterm religious fasting did not negatively affect iron status fasters ↑ ferritin levels and ↓ total iron-binding capacity, especially females	[26]
	120 Greek adults were followed longitudinally (60 fasters, 60 non-fasters)	1 year	no differences for other vitamins or minerals, before and after fasting, except for vitamin B2 ↓ calcium intake during fasting	[4]
	50 Athonian monks (mean age: 38.7 ± 10.6 years) and 43 males from the general population that regularly fast (20–45 y of age) and 57 age-matched Athonian monks	Restrictive and non-restrictive fasting days	↓ vitamin D levels and ↑ PTH with normal serum calcium levels	[15,16]
	37 strict fasters (18 males, 19 females, mean age 43.0 ± 13.1 years), and 48 age- and sex-matched controls (21 males, 27 females; mean age 38.6 ± 9.6 years)	40 days Measurements before and near completion of the Christmas fasting	↓ vitamin A & E levels during the fasting period for fasters These changes were related to changes in total cholesterol Vitamin E levels were correlated with changes in LDL and total cholesterol/HDL ratio	[5]
	60 Greek Orthodox participants, 30 with dyslipidemia and 30 without dyslipidemia, who followed the Greek Orthodox fasting	7 weeks	In both groups: ↓ vitamin B12 and calcium levels	[20]

↓*: Decrease; ↑**: Increase.

Considering the impact of fasting on antioxidant vitamins A and E status, a study conducted on strict fasters, mainly priests and nuns (18 males, 19 females, mean age 43.0 ± 13.1 years old), and 48 age- and sex-matched controls (21 males, 27 females; mean age 38.6 ± 9.6 years old) whose diet was evaluated before and at the end of the Christmas fasting period [5]. Both groups had good levels of vitamins A and E, and fasters had higher baseline vitamin levels than controls. However, their levels were reduced during the fasting period, while their levels increased for the controls [5]. The changes in serum vitamin levels during the fasting period were significantly related to changes in total cholesterol, whereas vitamin E levels were also correlated with changes in LDL and total cholesterol/HDL fraction [5]. On the other hand, another study that utilized 3-day food recall interviews showed that those who fasted had low intakes of most vitamins and elements, yet their biochemical indices were not affected [27].

Consequently, energy intake decreases during fasting, yet diet quality is better, with higher folate, iron, and magnesium intakes, as well as higher fiber and lower saturated and trans fatty acid intakes. Intake of calcium and vitamins D and B2 are a concern, nonetheless, while the intakes of most vitamins and elements seem to differ between study groups.

### 3.4. Headaches

Mitsikostas et al. [28] investigated the frequency of headaches among Athonian monks, who, apart from their dietary patterns, also have a different way of life and sleep program. Of the participating monks, 8.68% did suffer from headaches, which is less frequent than the general population [28]. The prevalence of migraine was 1.78%, tension headache 3.34%, and mixed headaches 1.87%, while cluster headache was not reported. Ninety percent of the monks who suffered from headaches had high scores on the Hamilton Scale for anxiety and depression (scored < 16). Interestingly, during the fasting periods, the frequency and intensity of headaches increased in the majority of monks [28].

In this respect, it should be noted that fasting or skipping meals are well-characterized migraine triggers. In fact, hypoglycemia, dehydration, caffeine withdrawal, free fatty acids, sympathetic nervous system activation, hypothalamic dysfunction, insulin, and several other hormonal factors have been considered potential triggers for headaches [29]. Moreover, a study conducted in Denmark’s general population found a lifetime prevalence rate of 4.1% for “fasting” headaches, which are usually diffuse or located in the frontal region, and the pain may be non-pulsating and of mild or moderate intensity [30]. In most cases, the headache appears after at least 16 h of fasting and resolves within 72 h after the resumption of food intake [31]. The likelihood of developing a “fasting” headache increases directly with the duration of the fast [30,31]. Headache sufferers have a higher risk of developing headaches during fasting than people who do not usually suffer from headaches [31]. Hypoglycemia and caffeine withdrawal have especially been implicated as causative factors, but the underlying mechanisms require further research [31].

### 3.5. Lifestyle and Mental Health

**Figure 5:**
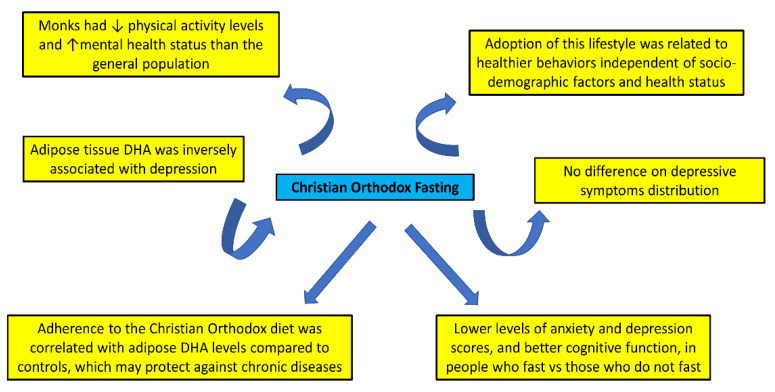
The potential beneficial effects of Christian Orthodox fasting on lifestyle and mental health (↑: Increase, ↓: Decrease).

Adherence to a healthy diet has been associated with better mental health across the lifespan [32,33,34,35,36]. The impact of Greek Orthodox fasting on mental health has not been thoroughly investigated, despite the impact of being religious on ethical values and mental health practices (e.g., alcohol use) (Table 4, Figure 5).

A cross-sectional study examined the Health-Related Quality of Life of Christian Orthodox Athonian monks and its correlation with demographic characteristics and a Sense of Coherence (SOC-14) [37]. One hundred sixty-six monks (45.5 ± 13.0 years old) from two monasteries and one skete participated in this study, and SF-12 and SOC-13 scales were completed by the monks, of whom 83.7% lived in communal monasteries, and the mean number of years as monks was 18.4 ± 12.1 [37]. Monks had lower physical activity levels (according to the Physical Component Summary score) than the general Greek male population, believed that their physical health was worse than the general public, but they had better mental health status (according to the Mental Component Summary score) [37]. It was documented that living in Mount Athos for a longer period and having a higher SOC score may have a protective role on the monks’ mental health [37].

In another study, semi-structured personal interviews were used to investigate a stratified sample of 20–65-year-old participants that followed the Greek Christian Orthodox lifestyle [36]. Adoption of this lifestyle was related to healthier behaviors (such as relaxation, life satisfaction, healthful nutrition, personal hygiene, and physical activity), independent of socio-demographic factors and health status [36]. In 24 strict fasters and 27 control participants with similar depressive symptom distribution, adipose tissue DHA was inversely associated with depression, while adherence to the Christian Orthodox diet was strongly associated with adipose DHA levels compared to controls, which may protect against chronic physical and mental diseases [37]. Moreover, another study found that middle-aged and elderly people who fast have lower levels of anxiety and depression scores, as well as better cognitive function, than those who do not fast. The authors did explore the diet the participants followed, and they did observe significant differences in dietary patterns between fasters and non-fasters [38]. Hence, the current data suggest a benefit of religious fasting on mental health and cognition.

## 4. Discussion

Christian Orthodox fasting is a “prudent” dietary pattern, low in refined carbohydrates and high in fiber and plant-derived foods. It has been studied over the last three decades and may contribute to overall human health. This type of dietary pattern may be of general interest concerning public health promotion. In particular, positive effects concerning glucose and lipid control have been observed, while there is still inconclusive evidence concerning blood pressure. Especially for lipid control, certain studies have indicated a positive impact of Orthodox religious fasting on total and LDL cholesterol. In this respect, it should be mentioned that several studies focused on the evaluation of the potential effects of Christian Orthodox fasting on the lipid profile of fasters.

As far as weight loss is concerned, fasters had lower body mass and lower caloric intake during the fasting periods. Even if there is a slight deficiency in some vitamins and micronutrients in some individuals, these could be prevented by the use of food supplements. These situations concern only people that already have deficiencies in certain nutrients. In the case of people with severe nutrient deficiencies, Christian Orthodox fasting should not be recommended, or it should be followed with the guidance of a registered dietitian/nutritionist.

In addition, despite lower calcium intake and lower consumption of dairy and soy products during fasting, older individuals adhering to Christian Orthodox fasting did not differ in bone mineral density, bone mineral content, or prevalence of osteoporosis from controls [39]. Thus, periodic abstinence from dairy and animal products generally does not seem to compromise bone health in older individuals [39].

Diet quality is improved during fasting, as shown by the participants’ food diaries, food frequency questionnaires, and 24-h recalls. The dietary practice of fasting is characterized by a diet high in complex carbohydrates and low in refined carbohydrates, with higher fruit and vegetable consumption, consequently higher fiber intakes, as well as higher iron and folate intake, which have also previously been associated with better mental health. However, the intakes of some vitamins and trace elements may not be sufficient, although biochemical indices may not reflect the low intakes, except for hypovitaminosis D in monks.

Nevertheless, considering headaches, despite the fact that monks had lower headache prevalence, the aches may be increased during fasting. In general, a high-quality diet rich in antioxidants, such as the Mediterranean diet, may play a positive role in headache management [40]. As far as lifestyle factors and mental health are concerned, monks did present a better quality of life and mental health than the general population, while better mental health and cognitive function have been observed in people who regularly fast [28,29,30,31]. Additionally, the Mediterranean diet has been studied regarding its impact on mental health. More to the point, there is substantial evidence [41,42,43] to support the fact that the Mediterranean diet has a positive impact on mental health, especially depressive symptoms and remission of depression. However, the available data remain scarce regarding mental health and headaches.

Several studies have examined the health impact of Christian Orthodox fasting and have also reported positive effects on blood lipids [20,21,22,23,24]. Koufakis et al. [44] noted a caloric restriction during fasting, accompanied by a decrease in fat intake and an increase in carbohydrate and fiber intake. Improvements in blood lipid control have been noted throughout the studies, especially on total and LDL cholesterol levels, yet with inconsistent findings on HDL cholesterol. Also, the lower dietary intakes of vitamins D and B12 and minerals, especially calcium, were a concern [44].

The Christian Orthodox fasting diet pattern can be compared to a “plant-based”, high-fiber diet, such as a pescatarian or a flexitarian diet. Pescatarians follow a vegetarian diet, yet they eat fish. Flexitarians consume no processed meat, low amounts of red meat and free sugars, moderate amounts of poultry, dairy, and fish, and high amounts of fruits, vegetables, legumes, and nuts [45,46]. Derbyshire et al. [47] evaluated 25 studies concerning the role of adherence to a flexitarian diet on health, specifically on body weight, cancer, diabetes and metabolic syndrome, and diet quality. The authors did find that such diet patterns may be related to weight loss and better metabolic health, reduced diabetes risk and hypertension, while it may possibly help patients with inflammatory bowel disease due to the high fiber intake [47]. Additionally, according to another report on sustainable nutrition, it was noted that adherence to a flexitarian diet was associated with a 19% lower incidence of premature mortality [48]. However, another study highlighted that adherence to a vegetarian or flexitarian diet may be associated with a greater risk of eating disorders, yet the results of the existing studies are conflicting [46]. Considering vegetarian diets, Tonstad et al. [49] examined the health effects of different types of vegetarian diets and found a protective effect against type 2 diabetes for pescatarian and semi-vegetarian diets, compared to non-vegetarian diets [49].

Moreover, Christian Orthodox fasting practices are more relaxed than other religious fasting practices yet more strict than others regarding the foods that are prohibited. Due to the prohibitions, fasters are led to follow a more prudent diet, yet the quality and characteristics of the dietary pattern may differ among fasters. In order to control for this, most studies recorded food intake with food diaries, 24 h recalls, and food frequency questionnaires during fasting and during regular days so as to compare fasting and non-fasting days.

However, several of the available clinical human studies have certain limitations and disadvantages. Most of them were conducted on a small sample size, which could not be generalized to the general population. Moreover, due to the methodology, recall bias is a possibility since several potential risk factors were self-reported by participants. Thus, no conclusions about causality can be made due to the design of most of the studies. The results of the existing clinical human studies cannot also be generalized beyond the Greek population or in other Caucasian populations of other ethnicities since most of them included Greek people with the same religion and traditional dietary pattern (the Mediterranean diet).

Moreover, it should be noted that BMI was utilized to distinguish fasters’ overweight and obesity status. Nevertheless, direct measures of body fat mass and distribution are required to extend and verify the existing results. In addition, most of the studies did not adjust for potential confounding factors to assess if Christian Orthodox fasting is independently associated with the reported beneficial effects on human health. On the other hand, the currently available studies have the advantage that they have evaluated for the first time a unique dietary pattern, such as Christian Orthodox fasting, which could be easily followed by any individual.

Dietary guidelines around the globe do focus on a healthy, varied, inclusive diet, with emphasis on plant-derived foods, and low in refined carbohydrates that could be compared to both the flexitarian or Mediterranean Diet, as well as Christian Orthodox fasting [39,50,51,52] in order to prevent physical and mental illness. Religious fasters follow this more traditional dietary pattern year-round for years. Hence, the benefits of adhering to this dietary pattern can be sustained long-term [53].

## 5. Conclusions

Overall, this prudent dietary pattern, as well as the lifestyle that accompanies Christian Orthodox fasting, could contribute to human health promotion and disease prevention. The impact of the carbohydrate content of this pattern on health measures and the gut microbiota needs to be explored, as there is a literature gap. Further studies are recommended on the impact of long-term religious fasting on HDL cholesterol levels and blood pressure, while the use of this periodic vegetarianism should be further studied as a measure of medical nutrition co-treatments in patients with chronic diseases, such as diabetes, cardiovascular disease and hypertension, as well as depression and anxiety. The importance of mental health in relation to Christian Orthodox fasting should also be further explored. In addition, further studies conducted on larger sample sizes from different countries and different ethnicities beyond Caucasians are strongly recommended, taking into consideration potential confounding factors by using multivariate regression analysis.

## Figures and Tables

**Table 2:** Studies concerning the impact of Christian Orthodox fasting on weight control.

Study Population	Study Period	Main Results	References
43 males from the general population that regularly fast (20–45 y of age) and 57 age-matched Athonian monks	Data were collected during both a restrictive and a non-restrictive day	Monks had lower BMI and lower body fat mass	[16]
36 (25 women & 11 men) monks from 5 Greek monasteries	40 days	Fasting led to a decrease in weight, upper arm circumference and triceps skinfold thickness	[18]
10 Greek Christian Orthodox monks aged 25–65 years, with BMI > 30 kg/m^2^	1 fasting week vs. non-fasting week Measurements on Palm Sunday week (fasting) and the week following Pentecost Sunday (non-fasting)	NS ↓* on monks’ body mass	[19]
120 Greek adults were followed longitudinally (60 fasters, 60 non-fasters)	1 year	Fasters had a ↓ 1.5% BMI than non-fasters at the end of the fasting period, while a ↓ 1.4% on BMI was in fasters after the fasting period	[17]

↓*: Decrease.

**Table 4:** Studies concerning the impact of Christian Orthodox fasting on well-being.

Study Population	Main Results	References
166 monks (mean age 45.5 ± 13.0 years) from two monasteries and one skete	Monks had ↓* physical activity levels than the general Greek male population and believed that their physical health was worse than the general public, but they had ↑** mental health status	[35]
20–65-year-old people who followed the Greek Christian Orthodox lifestyle	Adoption of this lifestyle was related to healthier behaviors independently of socio-demographic factors and health status	[36]
24 strict fasters and 27 controls	No difference in depressive symptoms distribution, adipose tissue DHA was inversely associated with depression, adherence to the Christian Orthodox diet was correlated with adipose DHA levels compared to controls, which may protect against chronic diseases	[37]
105 fasters and 107 non-fasters	Lower levels of anxiety and depression scores and better cognitive function in people who fast vs. those who do not fast	[38]

↓*: Decrease; ↑**: Increase.

## Data Availability

Not applicable.
